# Vitamin D and risk of hypertension among Black women

**DOI:** 10.1111/jch.14615

**Published:** 2023-01-06

**Authors:** Shanshan Sheehy, Julie R. Palmer, Yvette Cozier, Kimberly A. Bertrand, Lynn Rosenberg

**Affiliations:** ^1^ Slone Epidemiology Center Boston University Boston Massachusetts USA; ^2^ Boston University School of Public Health Boston Massachusetts USA

**Keywords:** Black American, hypertension, predicted vitamin D score, women

## Abstract

Evidence of an association between plasma 25‐hydroxyvitamin D [25(OH)D] levels and risk of hypertension, predominantly from studies of White individuals, suggests an inverse relationship. Limited data are available on Black individuals, who are more likely to have vitamin D deficiency. In the Black Women's Health Study (BWHS), a prospective study of 59 000 self‐identified Black women from across the US, we assessed levels of a validated predicted vitamin D score in relation to incident hypertension. We followed 42 239 participants who were free of cardiovascular disease and cancer from 1995 to 2019, during which time 19 505 incident cases of hypertension were identified. Cox proportional hazards model were used to calculate multivariable‐adjusted hazard ratios (HRs) and 95% confidence intervals (CIs) for the association of predicted vitamin D with the risk of incident hypertension. In age‐adjusted analyses, there was a strong inverse dose‐response association between predicted vitamin D score and hypertension risk, with an HR of .66 (95% CI: .63‐.68, p trend < .0001) for the highest quartile of predicted vitamin D relative to the lowest. After control for potential confounders including body mass index, physical activity, and cigarette smoking, the HR was attenuated to .91 (95% CI: .87–.95, p trend = .002). In this prospective cohort study of Black women, predicted vitamin D score was weakly inversely associated with the incidence of hypertension. This observed association may reflect an inability to fully control for confounding factors.

## 1 INTRODUCTION

Black Americans have an earlier onset and a higher incidence of hypertension, and have the highest rate of complications and deaths related to hypertension compared to other racial/ethnic groups.^1^, ^2^, ^3^ In particular, Black women in the U.S. have the highest hypertension prevalence (57%).^3^, ^4^ Evidence from in vitro studies, animal studies, and clinical trials suggests that vitamin D regulates blood pressure (BP) by inhibiting renin‐angiotensin‐aldosterone (RAAS) system activity, reducing vascular oxidative stress, and modulating the function of the vascular wall.^5^, ^6^, ^7^ Some evidence from epidemiological studies has suggested that lower plasma 25‐hydroxyvitamin D [25(OH)D] levels may be associated with a higher risk of incident hypertension and cardiovascular events.^8^, ^9^, ^10^, ^11^, ^12^, ^13^, ^14^, ^15^

Black Americans on average have lower circulating levels of vitamin D than Whites.^16^, ^17^, ^18^, ^19^, ^20^, ^21^ In 2011, the Institute of Medicine reported that 54% of Black Americans had vitamin D levels < 16 ng/ml (40 nmol/l), as compared to 27% of Hispanic and 10% of White Americans.^22^ Darker skin pigmentation among Black Americans results in lower production of vitamin D3 in the skin.^23^, ^24^ In addition, Black Americans generally consume less vitamin D from dietary sources than White individuals.^25^ Vitamin D deficiency may be associated with hypertension risk by activating the RAAS system^7^ and increasing renin and aldosterone levels.^26^ Aldosterone results in a greater increase in blood pressure in Black than White individuals.^26^, ^27^

Prior studies of vitamin D and hypertension have been focused on White populations and have yielded inconsistent findings.^16^, ^28^, ^29^, ^30^, ^31^, ^32^, ^33^ Only a few studies report results from Black individuals, who have disproportionately higher burdens of vitamin D deficiency and hypertension. The VITamin D and OmegA‐3 TriaL (VITAL), the first randomized intervention trial with la arge number of Black Americans (*n* = 5106), found that Vitamin D did not reduce risk of major CVD events in any racial subgroup, but the trial has not published results on risk of hypertension.^34^, ^35^ A cross‐sectional study found an association between low vitamin D and higher prevalence of hypertension among White but not Black participants.^36^ A small randomized trial found that vitamin D supplementation for 3 months was associated with modestly lower systolic pressure among Black participants.^37^

In the present study, based on 24 years of data collection in the Black Women's Health Study (BWHS), we assessed the relation between a validated predicted vitamin D score and hypertension risk. We hypothesized that higher predicted vitamin D score is associated with lower risk of hypertension.

## 2 METHODS

### 2.1 Study population

The BWHS is a prospective cohort study of 59 000 self‐identified Black women enrolled from across the United States in 1995.^38^ At baseline in 1995, participants were 21–69 years of age, with a median age of 38. Participants have provided information on biennial questionnaires regarding demographic characteristics, lifestyle and socioeconomic factors, and medical conditions. Follow‐up has been successful for 85% of potential person‐years through 11 completed biennial rounds of follow‐up. All but 5% of BWHS participants were born in the US.

The analytic sample consisted of 42 239 women after exclusion of 9294 women with prevalent hypertension at baseline in 1995, 563 women with cardiovascular disease at baseline, 482 women with cancer at baseline, and 6395 women for whom predicted vitamin D score could not be calculated due to missing data on predictors for the vitamin D predicted score.

The Boston University Medical Campus Institutional Review Board approved the study.

### 2.2 Exposure

The primary exposure was predicted vitamin D score, which was developed and validated against directly measured levels of serum vitamin D in the BWHS.^39^ In addition to maximizing sample size for statistical analysis, using a predicted vitamin D score can overcome drawbacks of prior studies that used a single measure of vitamin D. Serum vitamin D levels vary over time and are influenced by many factors, including season, age, body mass index (BMI), recent sun exposure, skin pigmentation, and dietary intake.^40^ Studies that use a single vitamin D measure cannot take that variation into account. Case‐control studies using serum vitamin D as an exposure could have the additional problem of reverse causation. A predicted vitamin D score in a follow‐up study with repeated data collection can be derived for multiple longitudinal time points, thus considering changes in lifestyle and environmental factors that may not be captured in a single blood sample.^40^

The predicted vitamin D score was developed and validated against directly measured levels of plasma 25(OH)D in 2856 BWHS participants.^39^ This validated predicted score of 25(OH)D has been inversely associated with risk of breast cancer and colorectal cancer in studies of BWHS participants.^39^, ^41^ The score was computed for each participant at each questionnaire cycle based on their values for the predictors at that point in time. Predictors include vitamin D supplementation, multivitamin use, BMI, dietary vitamin D intake, postmenopausal hormone use, vigorous physical activity, alcohol consumption, smoking, and oral contraceptive use. The correlation coefficient between predicted and measured plasma 25(OH)D was .49 (SD = .026). Vitamin D supplementation was the strongest predictor and accounted for 10% of the total variation. For our primary analyses, we modeled the predicted vitamin D score as a cumulative average^41^ and categorized predicted vitamin D score into quartiles. The cutoff points for predicted vitamin D score quartiles correspond to the cutoff points for quartiles of plasma 25(OH)D measured in the BWHS blood samples (21, 31, and 40 ng/ml for quartiles 2, 3, and 4, respectively).^39^

### 2.3 Outcome

Incident hypertension cases were identified through self‐reports on biennial questionnaires from 1995 through 2019. We considered a participant to have hypertension if she reported use of one or more antihypertensive medications or a physician diagnosis of hypertension together with diuretic use.^42^ In a validation study of self‐reported hypertension in the BWHS, 138 (99%) of 139 reports of hypertension for which we obtained medical records or physician checklists were confirmed, with all systolic pressures being 140 mmHg or higher and diastolic pressures being 90 mmHg or higher.^43^

### 2.4 Covariates

Hypertension risk factors to be controlled in the analysis were selected a priori, and included age (continuous, years), BMI (continuous, kg/m2), completed education (< 12, 13–15, 16, ≥17 years), geographic region (Northeast, South, Midwest, West), neighborhood socioeconomic status (SES, quintiles),^44^ physical activity level (continuous, hours of metabolic equivalent tasks per week based on both vigorous physical activity and walking),^45^ cigarette smoking (never smoker, past smoker <10 pack years, current smoker <10 pack years, past smoker 10+ pack years, current smoker 10+ pack years), history of type 2 diabetes (yes, no), and alcohol consumption (continuous, drinks/week).

For stratified analyses, we considered age (<50, 50–69, ≥70 years), BMI (<25, 25–29, ≥30kg/m2), diabetes (yes, no), smoking (never, ever), education (<12, 13–15, 16, ≥17 years), neighborhood SES (quintile 1, quintile 5), and Vit D supplement use (yes, no). All covariate information was based on information updated at each questionnaire cycle.

### 2.5 Statistical analyses

For our main analyses, we examined the relation between cumulative average predicted vitamin D score with risk of hypertension. We fit Cox proportional hazards models, stratified by age in 1‐year intervals and questionnaire cycle, with each participant contributing person‐years from baseline in 1995 until diagnosis of incident hypertension, death, loss to follow up, or end of follow up in 2019, whichever occurred first. Multivariable adjusted hazard ratios (HRs) and 95% confidence intervals (CIs), adjusted for the covariates listed above, were calculated comparing each quartile with the lowest quartile of predicted vitamin D score. We used the missing data indicator method for missing data in covariates. P for trend was tested using the median of the quartiles of predicted vitamin D score. We examined potential effect measure modification by age (<50, 50–69, ≥70 years), BMI (<25, 25–29, ≥30 kg/m2), diabetes (yes, no), smoking (never, ever), education (<12, 13–15, 16, ≥17 years), and neighborhood SES (quintile 1, quintile 5). Effect modification was evaluated using the Wald test.

## 3 RESULTS

During 583 296 person‐years of follow up, 19 505 incident cases of hypertension were identified. Compared with Black women in the lowest quartile of predicted vitamin D score at baseline, those in the highest quartile were younger, had a lower BMI, consumed less alcohol, were less likely to smoke, were more physically active, had higher education, and were more likely to live in a neighborhood with high SES (Table 1). Those in the highest quartile were also more likely to use vitamin D supplements.

**TABLE 1:** Age‐standardized baseline characteristics in Black Women's health study participants by quartiles of predicted vitamin D score

	Predicted vitamin D score			
	Q1	Q2	Q3	Q4
*n*	9774	10354	10848	11263
Age, year	38.4(9.3)	37.3(9.7)	36.7(9.8)	34.8(9.4)
Body Mass Index, kg/m^2^	31.4(7.2)	27.6(6.1)	25.9(5.0)	24.6(4.5)
Current alcohol use, drinks/week	2.8(6.1)	1.7(4.4)	1.2(3.4)	.8(3.0)
Smoking, pack years	4.9(9.3)	3.7(8.3)	3.1(7.6)	2.5(7.1)
Physical activity, MET hours/week	12.8(15.1)	16.4(16.4)	19.3(17.4)	24.3(17.9)
Type 2 diabetes, %	3.1	2.2	1.8	1.4
Education, %				
12 or less years	23.3	16.5	13.7	10.7
13–15 years	38.8	37.7	35.2	34.7
16 years	21.3	24.7	27.4	28.5
17+ years	16.3	20.9	23.6	26.0
Neighborhood SES: Highest quintile, %	14.8	17.6	21.4	23.5
Geographic region, %				
Northeast	25.3	27.9	28.0	28.7
South	31.1	30.8	30.1	31.3
Midwest	22.0	22.5	22.5	23.5
West	21.4	18.6	19.3	16.3
Current use of vitamin D supplement, %	.0	.08	.7	26.3

Values are means (± standard deviations) or percentages and are standardized to the age distribution of the study.

Abbreviations: MET, metabolic equivalent of task; Q, Quartiles; SES, Socioeconomic status.

There was an inverse dose‐response relationship between cumulative average predicted vitamin D score and risk of hypertension in analyses controlled only for age (Table 2). Compared to quartile 1 (the lowest predicted vitamin D score), HRs were .82 (95% CI: .79–.85), .72 (95% CI: .70–.75), and .66 (95% CI: .63–.68) for quartiles 2, 3, and 4, respectively (p trend < .0001, Table 2). Multivariable adjustment attenuated the associations: HRs were .98 (95% CI: .95–1.02), .98 (95% CI: .93–1.02), and .91 (95% CI: .87–.95) for quartiles 2, 3, and 4, respectively (p trend = .002, Table 2). BMI appeared to be the strongest confounder for this association. In the age adjusted model (Model 1), after adjustment in addition for BMI, HRs were .96 (95% CI: .92–.99), .93 (95% CI: .89–.97), and .85 (95% CI: .82–.89) for quartiles 2, 3, and 4, respectively. In the multivariate fully adjusted model, after further adjustment for supplemental vitamin D use, results remained similar. HRs were .98 (95% CI: .95–1.02), .98 (95% CI: .93–1.02), and .93(95% CI: .88–.98) for quartiles 2, 3, and 4). When restricting to participants with no use of supplemental vitamin D, there was no association between predicted vitamin D score with incident hypertension. HRs were .99 (95% CI: .95–1.03), .99 (95% CI: .94–1.03), .95 (95% CI: .89–1.01) for quartiles 2, 3, and 4, respectively (*p* = .14).

**TABLE 2:** Age‐ and multivariable‐adjusted hazard ratios for association of cumulative average predicted vitamin D score with risk of hypertension

Cumulative average predicted vitamin D					
	Quartile 1	Quartile 2	Quartile 3	Quartile 4	p trend
**Incident hypertension cases**	5174	5354	4842	4135	
**Person years**	116778	150534	160334	155650	
**Age‐adjusted HR (95% CI)** **^a^**	Ref	.82(.79, .85)	.72 (.70, .75)	.66 (.63, .68)	<.0001
**Multivariable‐adjusted HR (95% CI)** **^b^**	Ref	.98(.95, 1.02)	.98 (.93, 1.02)	.91 (.87, .95)	.002

P for trend was tested using the median of the quartiles of predicted vitamin D score.

Abbreviations: CI, confidence interval; HR, hazard ratio.

^a^ Stratified by age (continuous), questionnaire cycle.

^b^ Further adjusted for body mass index (continuous, kg/m2), educational level (<12, 13–15, 16, ≥17 years), geographic region (Northeast, South, Midwest, West), neighborhood SES (quintiles), physical activity level (continuous, hours of metabolic equivalent tasks per week), cigarette smoking (pack years of smoking, continuous, never smoker, past smoker <10 pack years, current smoker <10 pack years, past smoker 10+ pack years, current smoker 10+ pack years), history of diabetes (yes, no), current alcohol consumption (continuous, drinks/week).

In analyses stratified by age, BMI, smoking status, geographic region, education, neighborhood SES, and diabetes status, similar patterns to the overall association were observed (Table 3). No significant interactions were identified.

**TABLE 3:** Associations of predicted vitamin D with risk of hypertension by subgroup

	Ncases/py	Quartile 1	Quartile 2	Quartile 3	Quartile 4	*p* int
**Age, years**						
**<50**	11118/428425	Ref	.96 (.92, 1.01)	.93 (.88, .99)	.88 (.83, .93)	.11
**50**–**69**	7938/147689	Ref	1.01 (.95, 1.08)	1.02 (.95, 1.09)	.94 (.88, 1.00)	
**≥70**	449/7182	Ref	1.07 (.78, 1.46)	1.11 (.82, 1.53)	1.11 (.85, 1.45)	
**BMI, kg/m** ^2^						
**<25**	3299/198591	Ref	.92 (.82, 1.04)	.98 (.87, 1.10)	.94 (.84, 1.06)	.14
**25**–**29**	6637/200071	Ref	1.03 (.95, 1.12)	1.00 (.99, 1.02)	.91 (.83, .99)	
**≥30**	9569/184633	Ref	1.02 (.97, 1.08)	1.03 (.97, 1.10)	.95 (.89, 1.01)	
**Never smoking**	11829/398465	Ref	1.00 (.95, 1.05)	.99 (.93, 1.04)	.95 (.90, .99)	.83
**Ever smoking**	7676/184832	Ref	.97 (.91, 1.03)	1.01 (.94, 1.08)	.97 (.91, 1.03)	
**Neighborhood SES Q1**	3305/86730	Ref	1.02 (.93, 1.11)	1.04 (.94, 1.16)	.93 (.84, 1.03)	.88
**Neighborhood SES Q5**	3499/123227	Ref	.98 (.94, 1.03)	.98 (.94, 1.03)	.95 (.91, 1.00)	
**Diabetes**	2058/24490	Ref	.90 (.80, 1.01)	.89 (.78, 1.02)	.91 (.81, 1.03)	.85
**No diabetes**	17447/558806	Ref	1.00 (.96, 1.05)	1.01 (.96, 1.05)	.96 (.92, 1.00)	
**Geographic region**						
**Northeast**	4483/147573	Ref	1.04 (.96, 1.12)	1.01 (.92, 1.10)	.99 (.90, 1.07)	.93
**South**	7396/205914	Ref	.99 (.93, 1.06)	1.00 (.93, 1.07)	.96 (.90, 1.03)	
**Midwest**	4274/122775	Ref	.94 (.87, 1.02)	.95 (.86, 1.04)	.90 (.82, .98)	
**West**	3352/107034	Ref	.99 (.90, 1.08)	1.02 (.92, 1.13)	.95 (.86, 1.05)	
**Education**						
**<12**	3414/81145	Ref	1.04 (.95, 1.13)	1.02 (.92, 1.13)	1.03 (.93, 1.14)	.34
**13**–**15**	7148/210184	Ref	.96 (.90, 1.02)	.94 (.88, 1.01)	.89 (.83, .96)	
**16 years**	4696/158883	Ref	.98 (.91, 1.07)	1.00 (.92, 1.09)	.95 (.87, 1.03)	
**≥17 years**	4247/133084	Ref	1.02 (.93, 1.11)	1.06 (.96, 1.16)	1.01 (.92, 1.10)	

Model adjusted for age (continuous), questionnaire cycle (continuous), body mass index (continuous, kg/m^2^), educational level (< 12, 13–15, 16, ≥ 17 years), geographic region (Northeast, South, Midwest, West), neighborhood SES (quintiles), physical activity level (continuous, hours of metabolic equivalent tasks per week), cigarette smoking (pack years of smoking, continuous, never smoker, past smoker < 10 pack years, current smoker < 10 pack years, past smoker 10+ pack years, current smoker 10+ pack years), history of diabetes (yes, no), current alcohol consumption (continuous, drinks/week).

Abbreviations: CI, confidence interval; HR, hazard ratio; p int, p for interaction; py, person years.

## 4 DISCUSSION

In this prospective cohort study of 42 239 Black participants in the BWHS that included 19 505 cases of incident hypertension, there was a very weak association of predicted vitamin D score with risk of hypertension, with a HR of .91 (95%CI: .87–.95) for the highest quartile relative to the lowest. Because control for potential confounders such as BMI markedly reduced the HRs from what was observed in age‐adjusted analyses, it is possible the weak association remaining after such control is due to residual confounding from important risk factors.

Compared with Whites, Black individuals have lower serum vitamin D levels due to reasons that include darker skin pigmentation, genetics,^46^ diet, lifestyle and environmental factors.^47^ Current epidemiologic evidence on vitamin D and hypertension is predominantly based on studies of Whites. A meta analyses reported a RR of .88 (.81, .97) for incident hypertension per 10 ng/ml increment in baseline 25(OH)D levels.^32^ Comparing high versus low 25(OH)D level, the pooled relative risk was .70 (.58–.86) for risk of hypertension in another meta analyses.^9^ There is little evidence specifically for Black Americans.^16^, ^28^, ^29^, ^30^, ^31^, ^32^, ^33^ It is unclear as to the optimal dosage for cardiovascular health benefits from vitamin D^33^, ^35^ as well as race/ethnicity‐specific thresholds for vitamin D deficiency.^36^, ^48^ Evidence from randomized clinical trials failed to show a benefit of vitamin D supplementation on intermediate cardiovascular endpoints: there were no changes or only small reductions in blood pressure.^5^, ^31^, ^37^ However, the sample sizes in these trials were small, follow up was short, and vitamin D dosages differed. The VITAL trail, the first trial with a large number of Black participants (*n* = 5106), showed that Vitamin D did not reduce risk of major CVD events in any racial subgroup.^34^, ^35^ Results on hypertension were not reported separately. In a randomized clinical trial of 283 Black Americans, vitamin D supplementation for 3 months resulted in modestly lower systolic pressure.^37^ A cross‐sectional study found a null association between serum vitamin D and prevalence of hypertension among Black participants.^36^

Strengths of our study include the prospective cohort design, large sample size of Black women, large number of incident cases of hypertension, and the use of predicted vitamin D score to represent long‐term vitamin D status. The total of 19 505 incident cases of hypertension provided sufficient statistical power for this investigation. The 42 239 Black women in this study were from across the U.S with a wide range of ages, geographic distribution, and demographic characteristics. The vitamin D prediction score has been previously validated and shown to be associated with breast cancer and colorectal cancer incidence in the BWHS.^39^, ^41^ Compared with traditional studies using a single plasma vitamin D level, the predicted vitamin D score, derived repeatedly for multiple time points, represented long‐term exposures that a single blood sample cannot capture because of the relatively short half‐life of 25(OH)D.^39^, ^40^

Our study has several limitations. First, despite our effort to control for confounding factors, our study was observational in nature and still subject to unmeasured and residual confounding. While we can distinguish relatively low and high levels using the predicted vitamin D score, the distinction between deficient (<20 ng/ml) and insufficient (<30 ng/ml) in terms of measured plasma 25(OH)D level is uncertain because of the variability in the prediction model (prediction model R‐square = .25).^39^ Biologically, both vitamin D and parathyroid hormone level (PTH) influence calcium metabolism, and activation of the RAAS, and endothelial dysfunction.^49^ Vitamin D deficiency leads to elevated PTH and Black individuals have lower vitamin D and higher PTH levels compared with Whites.^49^ Observational studies suggest a synergistic association among Whites between vitamin D and PTH with risk of hypertension and cardiometabolic outcomes.^36^ In the BWHS, we did not collect information on PTH and therefore were not be able to assess the joint association of vitamin D and PTH. Renal function status can affect the relationship between vitamin D and hypertension. We did not have relevant information on renal function, such as eGFR and serum creatinine. Information on hypertension in the BWHS was self‐reported and did not allow for differentiation between essential hypertension and secondary hypertension. Since we did not have blood pressure measurements, we were unable to assess a possible dose response relationship with blood pressure, and were unable to examine the robustness of our results based on different definitions of hypertension (e.g., BP≥140/≥90 mmHg, vs. BP≥130/≥80 mmHg). Due to the asymptomatic nature of hypertension, the incidence of hypertension assessed in the BWHS is likely an underestimation of the actual incidence. However, given the prospective nature of this investigation, the measurement error in hypertension is likely to be non‐differential and unlikely to be correlated with the exposure (predicted vitamin D). Thus, the measurement error in hypertension may reduce statistical precision but is unlikely to introduce bias into our estimation.

In this large prospective investigation of predicted vitamin D and hypertension among Black women, predicted vitamin D score was very weakly inversely associated with incident risk of hypertension after control for confounding factors. Further studies on serum vitamin D levels are needed to assess the association of vitamin D with risk of hypertension in Black individuals. Measurements of vitamin D levels over time would be desirable, though very expensive to carry out. Some follow‐up studies currently in progress have the ability to determine new cases of hypertension, and the previous collection of blood samples could be leveraged for assays of serum vitamin D levels at the time of sample selection in relation to risk of hypertension subsequently.^50^ If an inverse association is confirmed going forward, then attention by physicians to consideration of “sufficient” levels of vitamin D among Black individuals will be desirable.

## AUTHORS CONTRIBUTION

Study concept and design: SS, JRP, LR. Acquisition of data: LR, JRP. Analysis and interpretation of data: SS, LR, JRP. Drafting of the manuscript: SS. Critical revision of the manuscript for important intellectual content: SS, LR, JRP, YZ, KAB. Statistical analysis: SS. Administrative, technical, or material support: LR, JRP. Study supervision: LR, JRP. SS had primary responsibility for final content. All authors have read and approved the final manuscript.

## CONFLICTS OF INTEREST

The authors have no conflict of interests. The sole role of the funders was to support data collection. The funders had no role in design and conduct of the study; collection, management, analysis, and interpretation of the data; preparation, review, or approval of the manuscript; and decision to submit the manuscript for publication.

## References

[1] Whelton PK , Einhorn PT , Muntner P , et al. Research needs to improve hypertension treatment and control in African Americans. Hypertension. 2016;68(5):1066‐1072. 27620388 10.1161/HYPERTENSIONAHA.116.07905 PMC5063700

[2] Wenger NK , Arnold A , Bairey Merz CN , et al. Hypertension across a woman's life cycle. J Am Coll Cardiol. 2018;71(16):1797‐1813. 29673470 10.1016/j.jacc.2018.02.033 PMC6005390

[3] Benjamin EJ , Blaha MJ , Chiuve SE , et al. Heart disease and stroke statistics‐2017 update: a report from the American Heart Association. Circulation. 2017;135(10):e146‐e603. 28122885 10.1161/CIR.0000000000000485 PMC5408160

[4] Ostchega Y , Fryar CD , Nwankwo T , Nguyen DT . Hypertension prevalence among adults aged 18 and over: United States, 2017–2018. NCHS Data Brief. 2020(364):1‐8. 32487290

[5] Al Mheid I, Quyyumi AA . Vitamin D and cardiovascular disease: controversy unresolved. J Am Coll Cardiol. 2017;70(1):89‐100. 28662812 10.1016/j.jacc.2017.05.031

[6] Yang T , Xu C . Physiology and pathophysiology of the intrarenal renin‐angiotensin system: an update. J Am Soc Nephrol. 2017;28(4):1040‐1049. 28255001 10.1681/ASN.2016070734 PMC5373463

[7] Forman JP , Williams JS , Fisher ND . Plasma 25‐hydroxyvitamin D and regulation of the renin‐angiotensin system in humans. Hypertension. 2010;55(5):1283‐1288. 20351344 10.1161/HYPERTENSIONAHA.109.148619 PMC3023301

[8] Ni W , Watts SW , Ng M , Chen S , Glenn DJ , Gardner DG . Elimination of vitamin D receptor in vascular endothelial cells alters vascular function. Hypertension. 2014;64(6):1290‐1298. 25201890 10.1161/HYPERTENSIONAHA.114.03971 PMC4430199

[9] Theodoratou E , Tzoulaki I , Zgaga L , Ioannidis JP . Vitamin D and multiple health outcomes: umbrella review of systematic reviews and meta‐analyses of observational studies and randomised trials. BMJ. 2014;348:g2035. 24690624 10.1136/bmj.g2035 PMC3972415

[10] Chowdhury R , Kunutsor S , Vitezova A , et al. Vitamin D and risk of cause specific death: systematic review and meta‐analysis of observational cohort and randomised intervention studies. BMJ. 2014;348:g1903. 24690623 10.1136/bmj.g1903 PMC3972416

[11] Qi D , Nie X‐L , Wu S , Cai J . Vitamin D and hypertension: prospective study and meta‐analysis. PloS One. 2017;12(3). 10.1371/journal.pone.0174298 PMC5373576 28358827

[12] Forman JP , Curhan GC , Taylor EN . Plasma 25‐hydroxyvitamin D levels and risk of incident hypertension among young women. Hypertension. 2008;52(5):828‐832. 18838623 10.1161/HYPERTENSIONAHA.108.117630 PMC2747298

[13] Cartier JL , Kukreja SC , Barengolts E . Lower serum 25‐Hydroxyvitamin D is associated with obesity but not common chronic conditions: an observational study of African American and Caucasian male veterans. Endocr Pract. 2017;23(3):271‐278. 27849379 10.4158/EP161456.OR

[14] Forman JP , Giovannucci E , Holmes MD , et al. Plasma 25‐hydroxyvitamin D levels and risk of incident hypertension. Hypertension. 2007;49(5):1063‐1069. 17372031 10.1161/HYPERTENSIONAHA.107.087288

[15] Zhang R , Li B , Gao X , et al. Serum 25‐hydroxyvitamin D and the risk of cardiovascular disease: dose‐response meta‐analysis of prospective studies. Am J Clin Nutr. 2017;105(4):810‐819. 28251933 10.3945/ajcn.116.140392

[16] Michos ED , Misialek JR , Selvin E , et al. 25‐hydroxyvitamin D levels, vitamin D binding protein gene polymorphisms and incident coronary heart disease among whites and blacks: the ARIC study. Atherosclerosis. 2015;241(1):12‐17. 25941991 10.1016/j.atherosclerosis.2015.04.803 PMC4466162

[17] Sakamoto R , Jaceldo‐Siegl K , Haddad E , Oda K , Fraser GE , Tonstad S . Relationship of vitamin D levels to blood pressure in a biethnic population. Nutr Metab Cardiovasc Dis. 2013;23(8):776‐784. 22770642 10.1016/j.numecd.2012.04.014 PMC3522760

[18] Alvarez JA , Gower BA , Calhoun DA , et al. Serum 25‐hydroxyvitamin D and ethnic differences in arterial stiffness and endothelial function. J Clin Med Res. 2012;4(3):197‐205. 22719806 10.4021/jocmr965w PMC3376878

[19] Fiscella K , Winters P , Tancredi D , Franks P . Racial disparity in blood pressure: is vitamin D a factor? J Gen Intern Med. 2011;26(10):1105‐1111. 21509604 10.1007/s11606-011-1707-8 PMC3181312

[20] Harris SS . Does vitamin D deficiency contribute to increased rates of cardiovascular disease and type 2 diabetes in African Americans? Am J Clin Nutr. 2011;93(5):1178s. 10.3945/ajcn.110.003491 21367947

[21] Rostand SG . Vitamin D, blood pressure, and African Americans: toward a unifying hypothesis. Clin J Am Soc Nephrol. 2010;5(9):1697‐1703. 20651156 10.2215/CJN.02960410

[22] Medicine Instituteof . Dietary Reference Intakes for Calcium and Vitamin D. National Academies Press; 2011. 21796828

[23] Wolf ST , Kenney WL . The vitamin D‐folate hypothesis in human vascular health. Am J Physiol Regul Integr Comp Physiol. 2019;317(3):R491. 31314544 10.1152/ajpregu.00136.2019 PMC6766707

[24] Harris SS . Vitamin D and African Americans. J Nutr. 2006;136(4):1126‐1129. 16549493 10.1093/jn/136.4.1126

[25] Moore CE , Murphy MM , Holick MF . Vitamin D intakes by children and adults in the United States differ among ethnic groups. J Nutr. 2005;135(10):2478‐2485. 16177216 10.1093/jn/135.10.2478

[26] Pratt JH , Jones JJ , Miller JZ , Wagner MA , Fineberg NS . Racial differences in aldosterone excretion and plasma aldosterone concentrations in children. N Engl J Med. 1989;321(17):1152‐1157. 2677724 10.1056/NEJM198910263211703

[27] Saxena T , Ali AO , Saxena M . Pathophysiology of essential hypertension: an update. Expert Rev Cardiovasc Ther. 2018;16(12):879‐887. 30354851 10.1080/14779072.2018.1540301

[28] Zhang X , Tu W , Manson JE , et al. Racial/ethnic differences in 25‐hydroxy vitamin D and parathyroid hormone levels and cardiovascular disease risk among postmenopausal women. J Am Heart Assoc. 2019 Feb 19;8(4):e011021. 30764690 10.1161/JAHA.118.011021 PMC6405652

[29] Robinson‐Cohen C , Zelnick LR , Hoofnagle AN , et al. Associations of vitamin D‐binding globulin and bioavailable vitamin D concentrations with coronary heart disease events: the multi‐ethnic study of atherosclerosis (MESA). J Clin Endocrinol Metab. 2017;102(8):3075‐3084. 28472285 10.1210/jc.2017-00296 PMC5546864

[30] Paul S , Judd SE , Howard VJ , Safford MS , Gutiérrez OM . Association of 25‐hydroxyvitamin D with incident coronary heart disease in the reasons for geographic and racial differences in stroke (REGARDS) study. Am Heart J. 2019;217:140‐147. 31629964 10.1016/j.ahj.2019.08.011 PMC6861690

[31] Manson JE , Cook NR , Lee IM , et al. Vitamin D supplements and prevention of cancer and cardiovascular disease. N Engl J Med. 2019;380(1):33‐44. 30415629 10.1056/NEJMoa1809944 PMC6425757

[32] Kunutsor SK , Apekey TA , Steur M . Vitamin D and risk of future hypertension: meta‐analysis of 283,537 participants. Eur J Epidemiol. 2013;28(3):205‐221. 23456138 10.1007/s10654-013-9790-2

[33] Pittas AG , Chung M , Trikalinos T , et al. Systematic review: vitamin D and cardiometabolic outcomes. Ann Intern Med. 2010;152(5):307‐314. 20194237 10.1059/0003-4819-152-5-201003020-00009 PMC3211092

[34] Bassuk SS , Manson JE , Lee IM , et al. Baseline characteristics of participants in the VITamin D and OmegA‐3 TriaL (VITAL). Contemp Clin Trials. 2016;47:235‐243. 26767629 10.1016/j.cct.2015.12.022 PMC4818165

[35] Bassuk SS , Chandler PD , Buring JE , Manson JE . The VITamin D and OmegA‐3 TriaL (VITAL): do results differ by sex or race/ethnicity? Am J Lifestyle Med. 2021;15(4):372‐391. 34366734 10.1177/1559827620972035 PMC8299924

[36] Xia J , Tu W , Manson JE , et al. Combined associations of 25‐hydroxivitamin D and parathyroid hormone with diabetes risk and associated comorbidities among U.S. white and black women. Nutr Diabetes. 2021;11(1):29. 34531372 10.1038/s41387-021-00171-2 PMC8676147

[37] Forman JP , Scott JB , Ng K , et al. Effect of vitamin D supplementation on blood pressure in blacks. Hypertension. 2013;61(4):779‐785. 23487599 10.1161/HYPERTENSIONAHA.111.00659 PMC3775458

[38] Rosenberg L , Adams‐Campbell L , Palmer JR . The Black Women's health study: a follow‐up study for causes and preventions of illness. Journal of the American Medical Women's Association. 1972;50(2):56‐58. 7722208

[39] Palmer JR , Gerlovin H , Bethea TN , et al. Predicted 25‐hydroxyvitamin D in relation to incidence of breast cancer in a large cohort of African American women. Breast cancer research. 2016;18(1):86‐86. 27520657 10.1186/s13058-016-0745-x PMC4983060

[40] Giovannucci E , Liu Y , Rimm EB , et al. Prospective study of predictors of vitamin D status and cancer incidence and mortality in men. J Natl Cancer Inst. 2006;98(7):451‐459. 16595781 10.1093/jnci/djj101

[41] Barber LE , Bertrand KA , Petrick JL , et al. Predicted Vitamin D status and colorectal cancer incidence in the Black Women's health study. Cancer Epidemiol Biomarkers Prev. 2021;30(12):2334‐2341. 34620630 10.1158/1055-9965.EPI-21-0675 PMC8643345

[42] National High Blood Pressure Education P . In: The Seventh Report of the Joint National Committee on Prevention, Detection, Evaluation, and Treatment of High Blood Pressure. Bethesda (MD): National Heart, Lung, and Blood Institute (US); 2004. 20821851

[43] Cozier Y , Palmer JR , Horton NJ , Fredman L , Wise LA , Rosenberg L . Racial discrimination and the incidence of hypertension in US black women. Ann Epidemiol. 2006;16(9):681‐687. 16458539 10.1016/j.annepidem.2005.11.008

[44] Bethea TN , Palmer JR , Rosenberg L , Cozier YC . Neighborhood socioeconomic status in relation to all‐cause, cancer, and cardiovascular mortality in the Black women's health study. Ethn Dis. 2016;26(2):157‐164. 27103765 10.18865/ed.26.2.157 PMC4836895

[45] Sheehy S , Palmer JR , Rosenberg L . Leisure time physical activity in relation to mortality among African American women. Am J Prev Med. 2020;59(5):704‐713. 32891468 10.1016/j.amepre.2020.05.013 PMC7577941

[46] Engelman CD , Fingerlin TE , Langefeld CD , et al. Genetic and environmental determinants of 25‐hydroxyvitamin D and 1,25‐dihydroxyvitamin D levels in Hispanic and African Americans. J Clin Endocrinol Metab. 2008;93(9):3381‐3388. 18593774 10.1210/jc.2007-2702 PMC2567851

[47] Hansen JG , Tang W , Hootman KC , et al. Genetic and environmental factors are associated with serum 25‐hydroxyvitamin D concentrations in older African Americans. J Nutr. 2015;145(4):799‐805. 25716552 10.3945/jn.114.202093 PMC4381765

[48] Zhang X , Tu W , Manson JE , et al. Racial/ethnic differences in 25‐Hydroxy vitamin D and parathyroid hormone levels and cardiovascular disease risk among postmenopausal women. J Am Heart Assoc. 2019;8(4):e011021. 30764690 10.1161/JAHA.118.011021 PMC6405652

[49] Gutiérrez OM , Farwell WR , Kermah D , Taylor EN . Racial differences in the relationship between vitamin D, bone mineral density, and parathyroid hormone in the National Health and Nutrition examination survey. Osteoporos Int. 2011;22(6):1745‐1753. 20848081 10.1007/s00198-010-1383-2 PMC3093445

[50] Sonderman JS , Munro HM , Blot WJ , Signorello LB . Reproducibility of serum 25‐hydroxyvitamin d and vitamin D‐binding protein levels over time in a prospective cohort study of black and white adults. Am J Epidemiol. 2012;176:615‐621. 22975199 10.1093/aje/kws141 PMC3530372

